# META-pipe Authorization service

**DOI:** 10.12688/f1000research.13256.2

**Published:** 2018-06-01

**Authors:** Inge Alexander Raknes, Lars Ailo Bongo

**Affiliations:** 1 Department of Chemistry, UiT - The Arctic University of Norway, Tromsø, 9037, Norway; 2 Department of Computer Science, UiT - The Arctic University of Norway, Tromsø, 9037, Norway

**Keywords:** ELIXIR AAI, SAML, OAuth 2.0, Authorization, Authentication

## Abstract

We describe the design, implementation, and use of the META-pipe Authorization service. META-pipe is a complete workflow for the analysis of marine metagenomics data. We will provide META-pipe as a web based data analysis service for ELIXIR users. We have integrated our Authorization service with the ELIXIR Authorization and Authentication Infrastructure (AAI) that allows single sign-on to services across the ELIXIR infrastructure. We use the Authorization service to authorize access to data on the META-pipe storage system and jobs in the META-pipe job queue. Our Authorization server was among the first SAML2 service providers  that integrated with ELIXIR AAI. The code is open source at: https://gitlab.com/uit-sfb/AuthService2.

## Introduction

ELIXIR brings together and coordinates European life science resources, including databases, software tools, training materials, cloud storage, and supercomputers. One of the resources developed in the ELIXIR-EXCELERATE project is META-pipe ^1^, an automated pipeline for annotation and analysis of metagenomic and genomic sequence data that is targeted for marine metagenomics. We will provide META-pipe as a web based data analysis service for ELIXIR users.

The ELIXIR Compute Platform builds distributed cloud, compute, storage, and access services for the life-science research community. An important part of the cloud platform is the geographically distributed Authentication & Authorization Infrastructure ( ELIXIR AAI) that provides services for identification, authentication, and authorization of ELIXIR end users. We have integrated our META-pipe Authorization service with the ELIXIR AAI, such that our users can use the single sign-on used across all ELIXIR services. Most users can sign-on using their home institution credentials; the remaining users can use Google, LinkedIn, or ORCID credentials. Our Authorization server was among the first services that integrated with ELIXIR AAI.

In this paper, we describe the design, implementation, and use of the META-pipe Authorization service. It limits which services and users are authorized to access and modify META-pipe datasets and job results. It *authenticates* users using ELIXIR AAI, and it *authorizes* access to data on the META-pipe storage system and jobs in the META-pipe job queue. We therefore use it as ad-hoc authentication for our own services.

We designed the META-pipe authorization service with three main design goals:

1. *Keep it simple and stupid*; especially for creating new services.
2. *Separation of concerns*. Keep authentication and authorization separate from the other META-pipe backend services.
3. *Stable interface* to avoid doing the same work multiple times. We therefore use existing standards in the interaction between services as these are assumed to be reasonably defined and stable.

Our solution is an external application decoupled from the rest of our services. It contains all the integration code needed to integrate with an external authentication service such as ELIXIR AAI, and it can be further be improved independent of the other META-pipe services. We also wanted it to be simple enough such that our small development team could implement it from scratch. We follow standards so we can re-use existing libraries and use proven, stable interfaces.

Existing out-of-the-box solutions did not fulfill our requirements at the time of implementation. For example, OpenID Connect, Shibboleth, and Keycloack are large standards with many mandatory parts that we do not need for our service. We therefore chose standards, libraries, and tools that are simple and developer friendly, and that can easily be incorporated in any framework/programming language combination as our services use Scala and Go, and Java. Today, ELIXIR AAI provides an OpenID connect provider that is an OAuth2 authorization server. It may be possible to use it instead of our Authorization service. We believe our service have advantages in development and testing since it can act as a middle authenticator that is easy to import in JavaScript. It is also a simple out-of-box-solution that we believe is a good introduction to OAuth2 and ELIXIR AAI. However, even if we use libraries such as Spring Security, we still have a small code base that we must maintain, and we have some missing features found in for example Shibboleth.

The rest of this paper describes the design and implementation of the server, how it is used by end-users and our backend services, and it gives an overview of the standards and libraries we have used. Finally, we outline ongoing and future work.

## Methods

This section summarizes the implementation and usage of the META-pipe authorization service. Additional details are in The META-pipe Authorization service design document.

### Design

The authorization service fits into the context of the META-pipe backend that has two *Clients*: the web application and the Galaxy tool. In addition, the context has several services, including storage and job management services. We have integrated our Authorization server with the ELIXIR AAI, which provides user identities from the ELIXIR federated IDP for European educational and research institutions, Google, LinkedIn, and ORCID. Users therefore use the single sign on ELIXIR web interface, and we rely on ELIXIR AAI to maintain (the federated) user databases.

Since some external IDPs, such as the Norwegian Feide that is one of the federated ELIXIR AAI IDPs, requires single sign-out we needed a way to revoke tokens on a short notice. This requires a central service for validating tokens and is one of the reasons we chose to use *Token Introspection* over *JWT Bearer Tokens* ( RFC-7523).

**Figure 1.**
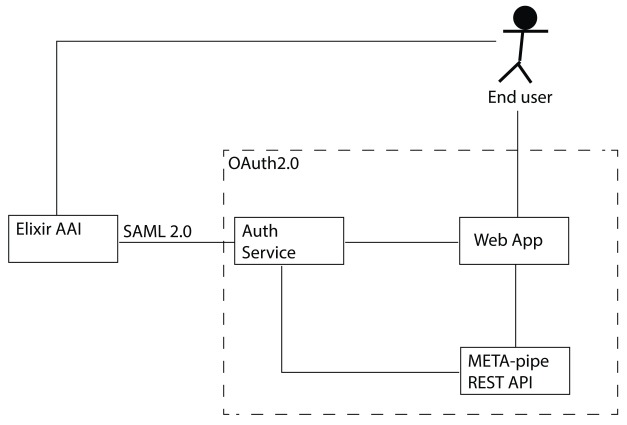
META-pipe Authorization service used by the META-pipe web application. The web application is an OAuth2 Client. The META-pipe REST API is the OAuth2 resource server, and it represents the META-pipe storage server and job manager server. The Authorization Service is an OAuth2 authorization server. The Authorization service is integrated with ELIXIR AAI. It is implemented to isolate the integration to the authorization server. We expose a REST API for the clients. The clients do not know about ELIXIR AAI.

The authorization server implements the *Authorization Code Grant* in the *OAuth 2.0 Authorization Framework* ( RFC-6749) ( Figure 1). The server uses *OAuth 2.0 Bearer Tokens* ( RFC-6750) as the access tokens since the *Bearer Tokens* are well supported by software libraries and they are required by the *Token Introspection* specification. In addition, the server implements an *OAuth 2.0 Token Introspection* ( RFC-7662) endpoint that the *Resource Servers* can use to introspect the tokens, whereby getting information about what can be accessed and if the token is still valid.

Our Meta-pipe web application stores the *Access Tokens* and *Refresh Tokens* in the browser's local storage since the tokens must be accessible from JavaScript. Using local storage is common, but it may expose our server to some cross-site attacks. We are therefore considering alternatives.

In OAuth 2.0 the *Access Token Scope* is defined by the Authorization server. Since we use a REST architecture for our services, each resource has its own URI. We have defined the *Access Token Scope* such that a *Resource Server* can determine if a request is authorized by comparing the *Access Token Scope* with the requested URI and method.

### Integration with ELIXIR AAI

We have integrated the META-pipe Authorization service with ELIXIR AAI. We use the *OAuth 2.0 Authorization Code Grant* since it is flexible enough to isolate the integration to the authorization server. We can therefore implement our *Clients* without them having to know neither about SAML nor ELIXIR AAI. We cannot use standards such as RFC-7522 ( *Security Assertion Markup Language (SAML) 2.0 Profile for OAuth2.0 Client Authentication and Authorization Grants*) and the more general RFC-7521 ( *Assertion Framework for OAuth 2.0 Client Authentication and Authorization Grants*), since these require the *Client* to directly interact with the *Issuer* and hence both the *Client* and the Authorization server need to know SAML and how to interact with the ELIXIR AAI. Another benefit of the *Authorization Code Grant* is that it has very good software library support.

A client using the *OAuth 2.0 Authorization Code Grant* redirects the *User Agent* to the *Authorization Endpoint* and then, after the *Resource Owner* has authorized the request, the *User Agent* is redirected to a URI defined by the *Client* where an *Authorization Code* is included in the URI query parameter. The *Client* can then exchange this *Authorization Code* for an *Access Token and a Refresh Token* by querying the *Token Endpoint*.

While RFC-6749 specifies how the *Client* interacts with the *Authorization Server*, it does not specify how the *Authorization Endpoint* obtains an authorization from the *Resource Owner*; this is left to the implementer of the *Authorization Endpoint*. Therefore, once the *User Agent* has been redirected to the *Authorization Endpoint* we can perform the authentication process that is required by the ELIXIR AAI (including any *Use Agent* interactions/redirects) if we are able to redirect the *User Agent* to the URI specified by the *Client* after the user has been authenticated.

### Integration with ELIXIR-Norway Galaxy/Feide

ELIXIR-Norway uses Galaxy as a common GUI for the analysis services provided for Norwegian users: https://nels.bioinfo.no/. The Galaxy users must be authenticated using the Feide authentication infrastructure ( Feide technical guide, Feide integration guide) that is used by all Norwegian universities, even if Feide is one of the federated IdP’s in ELIXIR AAI. We have integrated our ELIXIR-Norway Galaxy using an Apache HTTPD reverse proxy with AuthMemcookie and SimpleSAMLPHP. The Apache HTTPD proxies authenticated requests to the Galaxy web application with an HTTP header used to specify who is logged in.

The Galaxy tool has a user account on our Authorization Service, and it acts on behalf of all Feide users logged in Galaxy. The *Client* makes sure that Feide user data is protected from each other. The META-pipe Authorization service therefore does not directly rely on both Feide and ELIXIR AAI for user authentication. A Galaxy tool is defined by an XML file that describes how to launch a process and how the process' arguments should be presented in the Galaxy GUI. The tool developer maps every relevant parameter to a command line that Galaxy executes when the user runs the tool. The parameters include parameters selected by the user in the web GUI as well as a few contextual parameters, like the user's email address that is retrieved from Feide when the user authenticates.

### Software components coupling and limitations

The Authorization Server must know about ELIXIR AAI, the SAML protocol and the AAI specific attributes that provides information about a user. It must also have knowledge about which users are authorized to use which resources.

The Client must support *OAuth 2.0* ( RFC-6749) and *Bearer Token Usage* ( RFC-6750).

The Resource server must support *Bearer Token Usage* ( RFC-6750) and *OAuth 2.0 Token Introspection* ( RFC-7662). It must also know how to interpret a Scope in the correct context of the application.

### Implementation

We have implemented the authorization service in the Dropwizard web framework. Dropwizard is a lightweight Java web framework aimed at creating micro services. It uses Apache Oltu for handling the OAuth protocol, and it stores all its state in a PostgreSQL database using Hibernate ORM. Apache Oltu is a library for implementing OAuth 2.0 servers in Java. It manages the OAuth 2.0 protocol and helps serializing responses and parsing/validating requests. Hibernate is an Object Relational Mapper.

We have implemented the META-pipe web application ( *Client*) in JavaScript using the Client OAuth 2.0 library for interfacing with the authorization server and the Resource Servers. Client Oauth 2 (mulesoft/js-client-oauth2) is a JavaScript library for obtaining an authorization from an OAuth 2.0 Authorization server and for making authorized requests to a Resource Server. Client OAuth 2 supports several *Authorization Grants* including the *Resource Owner Password Credentials Grant* and *Authorization Code Grant*. It also supports *Bearer Token* usage ( RFC-6750). Client OAuth 2 can be used from a web browser or from within NodeJS.

*OAuth 2.0 Token Introspection* is not yet widely supported by software libraries, so we had to implement this from scratch. The specification is only 17 pages and it is very easy to implement. Our library implementation for *Resource Servers* is therefore only 176 lines of Go code.

### Standards

We here list the standards that we used and that are useful for others that are developing a similar service. These contain key definitions, abstraction, and representations:

- The OAuth 2.0 Authorization Framework ( RFC-6749) standard for performing authorization describes the *Acces Token*, which is a key abstraction in OAuth 2 that provides an abstraction layer, replacing different authorization constructs (such as username and password) with a single token understood by the resource server.
- The *The Oauth2.0 Authorization Framework: Bearer Token Usage* ( RFC-6750) defines the Bearer Token that is passed directly to the Resource Server when performing an authorized request.
- The *JSON Web token* (JWT) ( RFC-7519) standard describes a compact, URL-safe means of representing claims transferred between two parties.
- The *OAuth 2.0 Token Introspection* ( RFC-7662) defines a method for inspecting a token.

## Use cases

Here we describe the request flow for three use cases: META-pipe web application, command line, and Galaxy. In addition, we describe the request flow for the REST API used to submit jobs for these three uses cases, and we discuss limitation for downloading of META-pipe results using the browser.

The criteria for a user to be authorized to access the storage and compute resources needed to execute a META-pipe job is that the user has an account in one of the identify providers in the federated ELIXIR AAI. Once authorized the user will only have access to their own files and information about their own jobs. We plan to use the country of the user’s home institution to grant access to specific compute resources.

### User login via META-pipe web application

To authorize an end-user to run META-pipe analyses:

1. The end-user (Resource Owner) clicks “Log in with ELIXIR AAI” in the META-pipe web application that is implemented in JavaScript and therefore runs in the users browser.
2. The end-user is redirected to the Authorization Endpoint as defined by the Authorization Code Grant and then to the ELIXIR AAI using SAML web SSO.
3. The end-user logs in via one of the IDPs that are supported by the ELIXIR AAI and is then redirected back to the web application via the authorization server where it obtains an Authorization Code.
4. The web application obtains an Access Token by contacting the Token Endpoint with the Authorization Code.

### Command line tool

We provide a command line tool to a few power users so they can submit multiple jobs simultaneously. The tool obtains an Access Token by using the Client Credentials Grant.

### Galaxy tool login

When the META-pipe Galaxy tool ( *Client*) is called by Galaxy the end user will already be authenticated using the Galaxy/Feide integration at https://galaxy-uit.bioinfo.no/.

1. The Galaxy tool will get the user's email address as a command line parameter.
2. The Galaxy tool will be trusted to access all users’ data and will obtain an Access Token and a Refresh Token using the *Client Credentials Grant* ( RFC-6749) with a Scope that is limited to the requesting user's resources that are necessary to run a job.
3. The Galaxy Tool will hold the Access Token and Refresh Token in memory for subsequent API requests until the job has completed, and the tool terminates. No authorization state will be persisted between multiple tool invocations.

### API request to Resource Server

The above three use-cases use the META-pipe REST API to either submit jobs or monitor their progress. When a Client contacts an API endpoint (Resource Server) the following happens:

1. The Client provides the Bearer Access Token in the Authorization header to the API endpoint ( RFC-6750).
2. When the Resource Server receives the request, it will query the Introspection Endpoint to get the Scope of the Access Token and verify that it is still valid.
3. If the Access Token is valid the Resource Server will compare the Access Token' Scope (as returned by the Introspection Endpoint) to the scope required to process the request. If the request is authorized it will process the request, otherwise it will respond with an appropriate error code ( RFC-6750).

## Summary

We have described the design, implementation, and use of the META-pipe Authorization service. We use the Authorization service to authorizes access to data on the META-pipe storage system and jobs in the META-pipe job queue. Our Authorization server was among the first services that integrated with ELIXIR AAI.

### Future work

When downloading a data set (META-pipe result) from our storage service via the browser it is necessary to provide a direct link to the downloadable content while at the same time provide an Access Token to access the data. RFC-6750 allows for the use of an "access_token" URI query parameter containing a Bearer Token for authorization. We must use the URI since it is built by the Client after obtaining an access token from the Authorization service. This URI is used by the browser to download files. We do not have a better way to do this. A potential issue with this approach is that if a user shares a download link with other users they will get the user's access token. A solution to this is to limit the access token's Scope to only have read access to that particular resource, thus mitigating the risk of users inadvertently giving access to their account.

We plan to add an admin group for META-pipe administrators to a configuration file in the Authorization server. Another solution is to create a group in an Virtual Organization provided by ELIXIR AAI.

We would also like end-users to be able to add client credentials, when for example adding new users to the META-pipe Command Line Interface.

## Software and data availability

No data is needed to use the Authorization service.

The code is open source at: https://gitlab.com/uit-sfb/AuthService2.

Archived code at time of publication is: https://doi.org/10.5281/zenodo.1058995 ^2^

The software is licensed under The MIT License.

A user guide is at: https://gitlab.com/uit-sfb/AuthService2.
