# Supplementary material for: META-pipe Authorization service
Source: F1000Res. 2018 Jun 1;7:ELIXIR-32. Originally published 2018 Jan 9. [Version 2] doi: 10.12688/f1000research.13256.2 (PMC6008854; doi:10.12688/f1000research.13256.2)
Supplement: Peer review history

# Peer review: Referee response for version 2

**DOI:** 10.5256/f1000research.16449.r34601

**Published:** 2018-06-15

**Authors:** Mikael Linden

**Affiliations:** 1 CSC – IT Center for Science, Espoo, Finland

The authors have implemented OAuth2 authorisation server to manage user access to META-Pipe and to isolate the authentication and authorisation to a dedicated system component. The paper is a good presentation of the design.

I have read this submission. I believe that I have an appropriate level of expertise to confirm that it is of an acceptable scientific standard.

# Peer review: Referee response for version 2

**DOI:** 10.5256/f1000research.16449.r34600

**Published:** 2018-06-08

**Authors:** Frank Oliver Gloeckner

**Affiliations:** 1 Mi­cro­bial Ge­n­om­ics and Bioin­form­at­ics Re­search Group, Max Planck Institute for Marine Microbiology, Bremen, Germany

I guess it is now clear that at the time when META-pipe was developed an "from scratch" implementation of an Authentication system was necessary.

 If this holds true for now is a matter to be decided by the community.

 In any case META-pipe can be used as an good example.

I have read this submission. I believe that I have an appropriate level of expertise to confirm that it is of an acceptable scientific standard.

# Peer review: Referee response for version 1

**DOI:** 10.5256/f1000research.14381.r31273

**Published:** 2018-03-07

**Authors:** Frank Oliver Gloeckner

**Affiliations:** 1 Mi­cro­bial Ge­n­om­ics and Bioin­form­at­ics Re­search Group, Max Planck Institute for Marine Microbiology, Bremen, Germany

I agree with the authors that adding META-pipe to the ELIXIR AAI system is a useful invention. Since they were the first ones to add this feature to their pipeline I also appreciate the pioneering character of their work.

 To separate the META-pipe software from the authentication-server makes fully sense to me and supports the reusability of the authentication server for other projects.

 Nevertheless, I have two main questions/concerns where I did not find the answer in the manuscript:

  

1. Security: Although I am not an expert in this field I got the information that saving the Token in the local storage of the browser might be a risk. Since AAI is a security relevant component in every software system I would recommend consulting an expert in this field to check if the mode of implementation can be in general regarded as save.
2. From scratch implementation: It is rather unclear for me why the authors have decided to go for a “from scratch” implementation of their authorisation server? On page two they briefly state that existing out-of-the box solutions did not fulfil all requirements or are too heavy weight for their application, but only OpenID Connect is given as an example. From our experiences several open source authentication-servers exist, that can do the job pretty well e.g. Keycloak, GLUU, hydra, Shibboleth Identity Provider, with the last one is already used in the ELIXIR environment. To make the advantages of a new implementation clear I would recommend adding a paragraph and a table where the different servers are compared and the reasons why going for a fresh implementation are explained in detail. Sticking with a well-established implementation might have also had advantages with respect to potential security issues. A rather comprehensive list of SSO implementations can be found here https://en.wikipedia.org/wiki/List_of_single_sign-on_implementations.

I have read this submission. I believe that I have an appropriate level of expertise to confirm that it is of an acceptable scientific standard, however I have significant reservations, as outlined above.

# Response

**Published:** 2018-05-24

**Authors:** Lars Ailo Bongo

**Affiliations:** University of Tromso, Norway

Thank you for your comments and remarks. We have improved the paper as follows:

> *I got the information that saving the Token in the local storage of the browser might be a risk. Since AAI is a security relevant component in every software system I would recommend consulting an expert in this field to check if the mode of implementation can be in general regarded as safe.*

We could not find clear guidelines for the where to store the access token and refresh. It does not seem unusual to store the token in the browser local storage. We agree that this may expose our Authorization server to some attacks (such as cross-site attacks). We have therefore added a comment to the Design section that this may be unsafe, and we are considering alternatives for our server.

*> It is rather unclear for me why the authors have decided to go for a “from scratch” implementation of their authorisation server?*

We have added a discussion of the ELIXIR AAI Open ID Connect, Shibboleth, and Keycloak in the introduction. It was not an option when we started our implementation, but is clearly an alternative today. We still believe the simplicity of our server has some advantages for development and testing purposes. In addition, we use many libraries for the implementation such as Spring security. We also believe the simplicity, combined with this paper, makes it a good introduction to OAuth2 and ELIXIR AAI.

# Peer review: Referee response for version 1

**DOI:** 10.5256/f1000research.14381.r29637

**Published:** 2018-01-22

**Authors:** Mikael Linden

**Affiliations:** 1 CSC – IT Center for Science, Espoo, Finland

The paper is a useful description on how Meta-Pipe relies on OAuth2 framework for authorising access to the Meta-Pipe storage and job queue.

 What is the criteria the authorisation server uses to authorise the user to a certain resource? Based on what criteria is the authorisation granted to a user? Is it that users can only access their own files? Or are there also other criteria e.g. who in general can access the META-Pipe?

 ELIXIR AAI provides an OpenID Connect provider that is an OAuth2 authorisation server. Could you use it instead of developing your own server?

 Does the Meta-Pipe web application run in a user's browser or in a server?

 What is the relation of Feide and ELIXIR AAI in the context of Meta-Pipe? What is the relation of Meta-Pipe authorisation server and Feide? Does the Meta-Pipe authorisation server rely on them both for user authentication?

 In the end of Standards section on page 4 you refer to RFC 7522 as a spec you used but on the previous page you say you couldn't use that spec.

 You could improve figure 1 by clearly indicating where are the OAuth2 authorisation server, client and resource server.  Perhaps you could also introduce another drawing where the OAuth2 messages are presented. That would make it easier for the reader to follow how you have mounted OAuth2 for your deployment.

 Clients other than Meta-pipe Web app are missing in the Figure 1. What is the service behind the Meta-pipe REST API (from other sections I learn they are the job submission/management and storage services)?

 In the future work section; instead of displaying the user a URL with an embedded access token, why can't you protect the URI with OAuth2, triggering the client to obtain the Access token from the Authorisation server directly and then presenting it to the storage server.

I have read this submission. I believe that I have an appropriate level of expertise to confirm that it is of an acceptable scientific standard, however I have significant reservations, as outlined above.

# Response

**Published:** 2018-05-24

**Authors:** Lars Ailo Bongo

**Affiliations:** University of Tromso, Norway

Thank you for your insightful remarks. We have made the following improvements to our paper to address the raised concerns:

*> What is the criteria the authorisation server uses to authorise the user to a certain resource?*

*> Based on what criteria is the authorisation granted to a user?*

*> Is it that users can only access their own files?*

*> Or are there also other criteria e.g. who in general can access the META-Pipe?*

We added an explanation in the “Use cases” that all users with an ELIXIR AAI supported account have access to (only) their files and job information in META-pipe.

*> ELIXIR AAI provides an OpenID Connect provider that is an OAuth2 authorisation server. Could you use it instead of developing your own server?*

We added a discussion of the ELIXIR AAI Open ID Connect to the introduction. It was not an option when we started our implementation. We still believe the simplicity of our server has some advantages for development and testing purposes. For example as an introduction to OAuth2 and ELIXIR AAI.

*> Does the Meta-Pipe web application run in a user's browser or in a server?*

We now specify in Use Cases; User login via META-pipe web application that the web application is run only in the browser.

*> What is the relation of Feide and ELIXIR AAI in the context of Meta-Pipe?*

*> What is the relation of Meta-Pipe authorisation server and Feide? Does the Meta-Pipe authorisation server rely on them both for user authentication?*

We added explanations in Methods; Integration with ELIXIR-Norway Galaxy/ Feide that we must use Feide for Galaxy. We also describe how the Galaxy tool has an account on the Authorization service, and acts on behalf of all Feide users logged in Galaxy. The Authorization service therefore do not directly rely on both for user authentication.

*> In the end of Standards section on page 4 you refer to RFC 7522 as a spec you used but on the previous page you say you couldn't use that spec.*

It is correct that we do not use RFC 7522, so we have removed it from the list.

*> You could improve figure 1 by clearly indicating where are the OAuth2 authorisation server, client and resource server.  Perhaps you could also introduce another drawing where the OAuth2 messages are presented. That would make it easier for the reader to follow how you have mounted OAuth2 for your deployment.*

*> Clients other than Meta-pipe Web app are missing in the Figure 1. What is the service behind the Meta-pipe REST API (from other sections I learn they are the job submission/management and storage services)?*

We have added an explanation to the figure caption that the web application is an OAuth2 Client, the META-pipe REST API is the OAuth2 resource server, and the Authorization Service is an OAuth2 authorization server. We chose not to make changes to the figure since we try to keep it simple for illustration purposes, and since more detailed figures for the OAuth2 messages can be found in the standard documentation.

We also added a description to the figure caption that the META-pipe servers are represented by the REST API box.

*> In the future work section; instead of displaying the user a URL with an embedded access token, why can't you protect the URI with OAuth2, triggering the client to obtain the Access token from the Authorisation server directly and then presenting it to the storage server.*

*Misunderstanding? Is in line with OAuth2 specification.*

We added an explanation in Future Work that the URI is built after the client obtains an access token from the Authorization service. We do not know of a better way to do this.
